# Paracetamol (acetaminophen) intoxication and basal fatty tubular vacuolisation: a case-control study and review of the literature

**DOI:** 10.1007/s00414-026-03942-7

**Published:** 2026-08-01

**Authors:** Ahlström Stina, Tamsen Fredrik

**Affiliations:** 1 Department of Forensic Medicine, National Board of Forensic Medicine, Box 1024, Uppsala, SE-751 40 Sweden; 2 Department of Biomedical and Clinical Sciences, Science for Life Laboratory, Linköping University, Linköping, SE-581 85 Sweden; 3 Department of Surgical Sciences, Uppsala University, Akademiska sjukhuset, ingång 70, Uppsala, SE-751 85 Sweden

**Keywords:** Paracetamol (acetaminophen), Intoxication, Basal fatty tubular vacuolisation, Forensic diagnostics

## Abstract

Basal fatty tubular vacuolisation (BFTV) of the kidneys has mostly been associated with ketoacidosis. Its occurrence in paracetamol intoxication has not been described. Paracetamol in overdose is hepatotoxic and nephrotoxic but does not cause ketoacidosis. This study aimed at investigating whether BFTV occurs in paracetamol intoxication. The Swedish National Board of Forensic Medicine database was searched for the Uppsala area for 2016–2024 for paracetamol intoxication cases with kidney samples taken. Two equally-sized control groups, matched for age, sex and *post-mortem* interval, were selected from the same period: other intoxications and hangings. The kidney slides were evaluated by both authors for BFTV according to the scale “none-possible-scant-easily identifiable”. The kidney slides were also marked with ten fields and evaluated for the number of fields with BFTV and for the number of fields with formalin pigment deposition. A Kruskal-Wallis test was used to calculate significant differences between the groups. Spearman’s rho was used to evaluate associations and a p-value < 0.05 was considered statistically significant. The database search yielded 24 cases. Paracetamol intoxications showed a higher degree of BFTV and more BFTV-positive fields than both control groups (*p* < 0.05). The number of BFTV-fields correlated with the degree of BFTV and the number of fields with formalin pigment deposition (Spearman’s rho 0.691 and 0.544, respectively, *p* < 0.001). The correlation between the authors’ assessments was moderate. Thus, the present study found an association between paracetamol intoxication and BFTV. This likely represents a renal stress reaction due to paracetamol toxicity and not ketoacidosis.

**Supplementary Information:**

The online version contains supplementary material available at https://doi.org/10.1007/s00414-026-03942-7.

## Background

Basal fatty tubular vacuolisation (BFTV) refers to the accumulation of fatty vacuoles in the renal tubular cells [1, 2]. It should be separated from the Armanni-Ebstein lesion, which refers to renal tubular swelling by clear, glycogen-filled macrovacuoles in the context of poorly controlled diabetes [3, 4]. However, these two concepts are sometimes erroneously used synonymously [2].

BFTV has primarily been associated with diabetic ketoacidosis (DKA) [5, 6], alcoholic ketoacidosis (AKA) [7], and hypothermia [8, 9]. BFTV, usually to a moderate degree, has also been associated with diabetes [6], and alcohol use disorder (AUD) [7], without concomitant ketoacidosis, and to a minor extent with other conditions [10]. A histopathological confirmation that vacuoles in the tubules consist of fat requires a special fat stain [11], such as osmium or oil red, but formalin pigment deposition in the vacuoles is an indication that the vacuoles constitute fat [12, 13].

Thus, BFTV has primarily been associated with ketoacidotic conditions. However, within forensic pathologic case work, we observed that BFTV seemed to appear in cases of intoxication with paracetamol (referred to as acetaminophen in the United States [14]). BFTV in relation to paracetamol intoxication has not previously been described in the scientific literature, although Auld et al. mention one case of BFTV in a person with AUD and paracetamol toxicity, but without discussing a possible connection [7]. In normal use, paracetamol is metabolised in the liver to glucuronide or sulphate metabolites. A small amount is metabolised into a reactive metabolite, N-acetyl-p-benzoquinone imine (NAPQI) that is metabolised by hepatic gluthatione. However, in large doses or in persons with an underlying predisposing condition, the gluthatione reserve is exhausted, and NAPQI accumulates. NAPQI exerts its toxic effect on the mitochondria of the liver cells, ultimately causing liver necrosis and subsequent liver failure. Mitochondrial inhibition occurs before cellular damage develops [15–17].

In addition to liver toxicity, paracetamol may also cause acute kidney injury (AKI), characterised by acute tubular necrosis [18]. AKI may develop alongside the liver injury but it may also occur in isolation [19]. Histological studies of the kidneys in paracetamol intoxication are scarce. In a study on rats, Isik et al. described tubular epithelial vacuolisation in paracetamol overdoses [20] but they did not discuss the nature of the vacuolisation. Björck et al. studied renal biopsies from three patients with paracetamol intoxication. They described focal tubular epithelial cell necrosis but they did not mention any vacuolisation [21]. Both the aforementioned studies included black-and-white images of a histopathological specimen, but the print quality does not allow for an evaluation whether any basal tubular vacuolisation consistent with BFTV is present in the samples. Whether BFTV occurs in paracetamol intoxication is therefore unknown.

Based on our observations of BFTV in paracetamol intoxication, and based on the fact that paracetamol may cause acute kidney injury, we designed a retrospective study to investigate whether BFTV occurs in paracetamol intoxication. Since the paracetamol concentration by the time liver necrosis occurs may be low or undetectable [22], finding another possible indicator for paracetamol intoxication besides toxicological analysis would be of value. We also review the literature and propose an explanation why BFTV may develop in paracetamol intoxication.

## Materials and methods

The database of the Swedish National Board of Forensic Medicine was searched for the Uppsala area for the years 2016 through 2024 for autopsy cases in which either paracetamol and/or liver necrosis were mentioned on the death certificate and in which histological samples from the kidneys and the liver had been taken at the time of autopsy. Children under the age of 18 were excluded. The cases were further divided into single intoxications with paracetamol, and mixed intoxications with paracetamol as one substance.

Two equally sized control groups with samples taken from the kidneys and the liver at the time of autopsy were selected from the same time period from the Uppsala area. The first control group consisted of other intoxications (the cause of death either a single intoxication with one substance, or a mixed intoxication with more than one substance other than paracetamol). The second control group consisted of hanging deaths. The control groups were matched on a case level with the paracetamol group for age, sex and *post-mortem* interval (PMI). PMI was defined as the number of days between the date of death and the date of performing the autopsy. When the date of death had been assessed by the forensic pathologist as either certain or probably, the calculation was straight forward. If the date of death was neither certain nor probable, it was assessed to be in the midst of the interval between the dates of last seen and found dead. If either of these dates was also missing, an assessment of the probable date of death was made by one of the authors from the available background information and autopsy report.

The available background information (police reports and health care records) was checked for information on known AUD and diabetes for all three groups. The results from the forensic toxicology analyses were retrieved from the database for all groups.

**Fig. 1:**
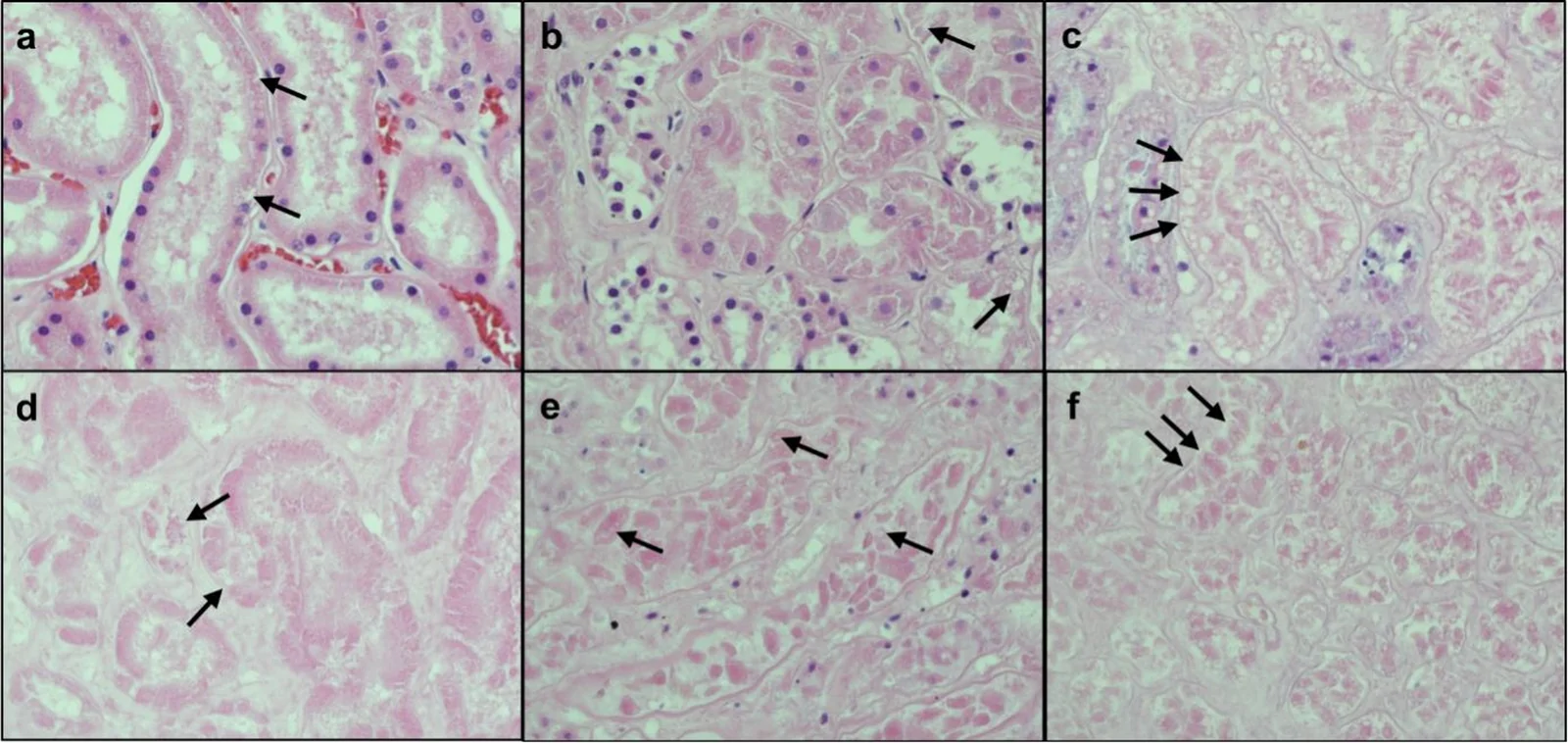
Representative histological images in 400x magnification showing the three BFTV-positive grading categories used in the study, a-c in non-putrefied tissue, d-f in putrefied tissue. (**a**, **d**) possible, (**b**, **e**) scant, (**c**, **f**) easily identifiable. Arrows indicate vacuoles. BFTV = Basal fatty tubular vacuolisation

Using Excel (Microsoft 365, version 2408), all cases were assigned an arbitrary ID-number. The formalin-fixed paraffin-embedded (FFPE) tissue samples from the kidney and the liver were retrieved from the archive. If more than one sample was available from either the liver or the kidneys, the sample with the lowest assigned number in the original case was selected. Thus, one kidney sample and one liver sample from each case was sectioned, and the new slides were prepared from these. The new slides were marked with the arbitrary number. The kidney slides were stained with haematoxylin-eosin (HE), and Periodic acid-Schiff (PAS) stain. The liver slides were stained with HE, Herovici, and Gordon stain. All samples were individually evaluated by both authors, who were blinded as to what group an individual slide belonged. The HE-stained kidney slides were marked with the microscope’s marking stamp with 10 individual fields, each with an area of 3.8 mm^2^, corresponding to the field of view at 100x magnification. The fields were evaluated using the 400x magnification on the microscope, for the presence of tubular vacuolisation suggestive of BFTV. The number of fields containing any BFTV was counted, rendering every slide a score between 0 and 10 (quotient scale). In addition, an overall estimation of the existence of BFTV in the entire slide was made, again using the 400x magnification, according to the scale “none-possible-scant-easily identifiable” (ordinal scale). In Fig. 1, examples of possible, scant, and easily identifiable BFTV are presented, both in tissue without and with putrefaction.

**Fig. 2:**
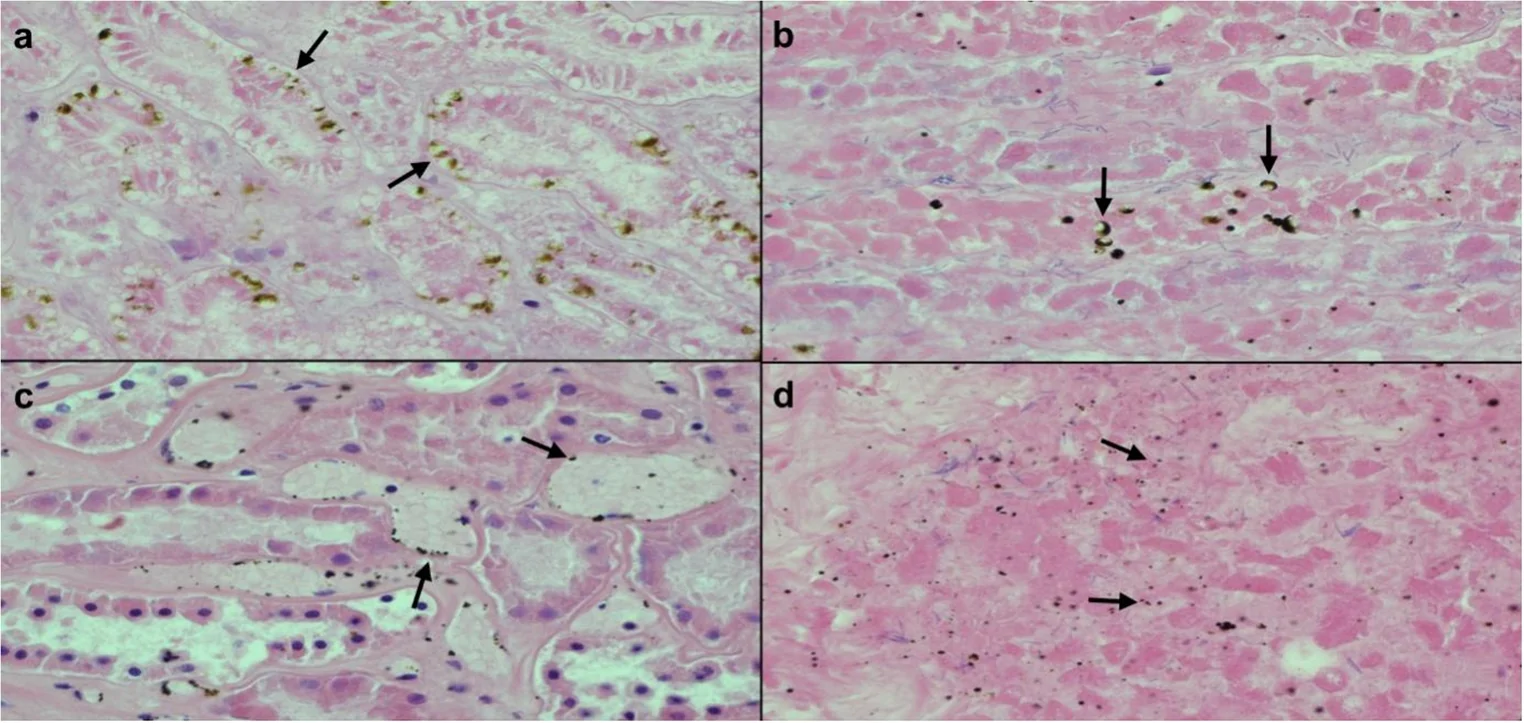
Histological images in 400x magnification where the upper row shows formalin pigment deposition in a pattern suggestive of BFTV in (**a**) non-putrefied and (**b**) putrefied tissue, and the lower row shows general formalin pigment deposition in (**c**) non-putrefied and (**d**) putrefied tissue. Arrows indicate formalin pigment deposition. BFTV = Basal fatty tubular vacuolisation

In order to estimate whether the observed vacuoles consisted of fat, a further evaluation of the 10 marked fields was made with the 400x magnification for the presence of formalin pigment deposition in a pattern suggestive of BFTV, see Fig. 2a-b. Thus, each slide again received a number between 0 and 10. In order to evaluate the significance of putrefaction, the kidney HE-slides were evaluated for degree of putrefaction according to the scale “minimal-slight-moderate-severe”. As a further marker for putrefaction, the HE-stained kidney slides were evaluated for the presence of general formalin pigment deposition (yes/no), regardless of the location of the depositions, see Fig. 2c-d. The PAS-stained kidney slide was similarly marked with 10 fields and each PAS-stained slide was evaluated for the number of PAS-positive fields. Concerning the liver, the slides were evaluated for liver necrosis (yes/no/cannot be determined) based on a combined evaluation of all three stains.

One of the author’s evaluations were used for the statistical analyses. Both authors evaluated all cases to calculate the inter-rater reliability.

### Statistics

**Fig. 3:**
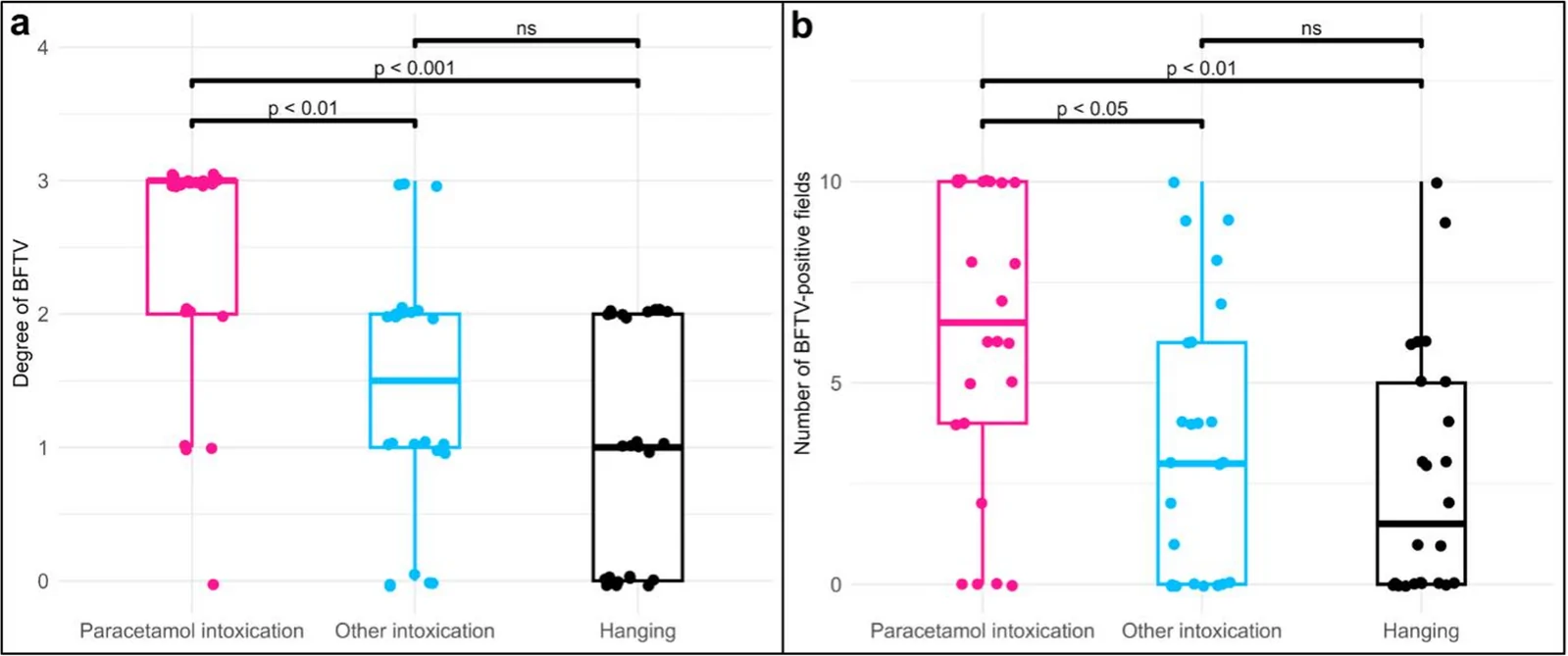
Results of the two BFTV-scales in the three different groups. The box-plots show median, inter-quartile range and range, the jitter represents individual cases. BFTV = Basal fatty tubular vacuolisation

Microsoft Excel (Microsoft 365, version 2408) was used for descriptive statistics, and SPSS (version 30.0.0.0) and R (version 4.3.1) for analytical statistics. Kruskal-Wallis was used to test for differences between the groups, with Dunn’s post-hoc test and Bonferroni’s correction for multiple comparisons. A p-value < 0.05 was considered statistically significant. Spearman’s rank correlation, or rho (ρ), was used to evaluate the correlation between the degree of vacuolisation and the number of BFTV-positive fields, between the degree of general formalin pigment deposition and the number of fields with formalin pigment deposition, between the number of fields with BFTV-suggestive formalin pigment deposition and degree of putrefaction, between the occurrence of general formalin pigment deposition and degree of putrefaction, between the number of fields with BFTV-indicative formalin pigment deposition and number of BFTV-positive fields, and between the number of fields with BFTV-indicative formalin pigment deposition and the degree of vacuolisation. Spearman’s rho was also used to evaluate the inter-rater reliability. Figure 3 was generated with R.

## Results

When performing the power calculation, the somewhat arbitrary assumption was made that cases would have a mean of 6 of 10 fields positive for BFTV, compared to 2 of 10 in the control groups. This rendered a minimum of 16 cases in each group for a power of 80%. During the study period, 30 cases were identified in the preliminary search. Of these, 6 cases were excluded due to not being related to paracetamol intoxication, leaving 24 cases for inclusions. The 24 paracetamol intoxication cases included 18 women and 6 men. Of these, 14 were mixed intoxications with paracetamol as one of the substances, and 10 were single intoxications with only paracetamol. In one case, alcoholic ketoacidosis was considered the underlying cause of death, and the paracetamol intoxication was considered contributing cause of death. In all other cases, the intoxication was considered the underlying cause of death.

The mean age of the paracetamol intoxication group was 56.9 years. The mean age of the first control group (other intoxications) was 56.8 years, and for the second control group (hangings) 55.8 years. The ages of the control groups did not significantly differ from the paracetamol cases or from each other. The PMI range for the paracetamol intoxications was between 1 and 15 days and mean 7 days, for the other intoxications between 1 and 14 days and mean 7 days, and for the hangings between 2 and 11 days and mean 6 days. The PMI did not significantly differ between the groups. For the paracetamol intoxication group, three cases lacked information for PMI calculation and had to be assessed by one of the authors. All control cases contained enough information for PMI calculation.

### Basal fatty tubular vacuolisation

Comparisons between the groups with respect to BFTV are presented in Fig. 3. The paracetamol intoxications displayed a significantly higher degree of BFTV compared to both other intoxications and hangings (*p* = 0.003 and < 0.001, respectively). The most common degree in paracetamol intoxications was “easily identifiable” (63% of the cases). Other intoxications were most often classified as “scant” (37.5% of the cases), while hangings were either “none” or “scant” (both 37.5%) in the majority of cases. The paracetamol intoxications had a mean number of 6.3 BFTV-positive fields, the other intoxications had a mean value of 3.5, and the hangings 2.7. The difference between the paracetamol cases and the two control groups was statistically significant (*p* = 0.029 and 0.003, respectively). The difference between the two control groups was not significant for either the degree of BFTV or for the number of BFTV-positive fields. The degree of BFTV and the number of BFTV-positive fields had a strong correlation (ρ = 0.691, *p* < 0.001). Both the degree of BFTV and the number of BFTV-positive fields correlated with the number of fields with BFTV-indicative formalin pigment deposition (ρ = 0.499 and 0.544, respectively, *p* < 0.001).

The number of fields with BFTV-indicative formalin pigment deposition correlated with general formalin pigment deposition (ρ = 0.464, *p* < 0.001), but no statistically significant correlation was found with the degree of putrefaction (ρ = 0.213, *p* = 0.072). General formalin pigment deposition had a weak but statistically significant correlation with the degree of putrefaction (ρ = 0.272, *p* = 0.021).

The inter-rater reliability for both the degree of BFTV and the number of BFTV-positive fields was moderate (ρ = 0.605 and 0.554, respectively, both with *p* < 0.001).

### The paracetamol intoxications

Of the 24 cases of paracetamol intoxication, paracetamol had been analysed in femoral blood in 22 cases. The median was 160 mg/L, the mean 247 mg/L, and the range 0.0 mg/L through 1110 mg/L. In two cases, femoral blood was not available, and the analysis of paracetamol had been performed in heart blood. The results in these two cases were 120 mg/L, and 88 mg/L, respectively. In two cases, paracetamol was not detected in femoral blood, but its part in the cause of death was based on background information. In cases of single intoxication with paracetamol, the mean paracetamol concentration was 187 mg/L femoral blood, the median 145 mg/L, and the range 0–500 mg/L. In the cases of mixed intoxications, the mean paracetamol concentration was 291 mg/L femoral blood, the median 160 mg/L, and the range 0–1100 mg/L.

The individual results of the BFTV assessments in the paracetamol intoxication cases are presented in Table 1. Ten cases were single intoxications with paracetamol, while the other 14 were mixed intoxications with paracetamol and one or more other substances. The other substances involved were mostly opioids, benzodiazepines, hypnotics, sedating antihistamines, anti-depressants and neuroleptics.

**Table 1:** Vacuolisation, formalin pigment deposition, putrefaction and intoxication type (single or mixed) in the paracetamol intoxications. BFTV = Basal fatty tubular vacuolisation

Degree of vacuolisation	Number of BFTV-positive fields	Number of fields with formalin pigment deposition	Degree of putrefaction	Intoxication with paracetamol as single substance, or in combination with others (mixed)
Easily identifiable	10	9	Moderate	Single
Easily identifiable	10	2	Moderate	Single
Easily identifiable	10	0	Severe	Mixed
Easily identifiable	8	7	Severe	Mixed
Easily identifiable	10	2	Severe	Single
Easily identifiable	4	8	Moderate	Mixed
Easily identifiable	10	10	Minimal	Mixed
Easily identifiable	5	4	Moderate	Single
Easily identifiable	10	10	Mild	Single
Easily identifiable	7	4	Severe	Mixed
Easily identifiable	2	10	Severe	Mixed
Easily identifiable	6	8	Moderate	Mixed
Easily identifiable	10	10	Moderate	Single
Easily identifiable	10	0	Moderate	Single
Scant	8	0	Minimal	Mixed
Scant	4	3	Moderate	Mixed
Possible	0	0	Severe	Mixed
Undetermined	0	0	Mild	Single
Possible	0	0	Severe	Mixed
Scant	6	6	Mild	Mixed
None	0	0	Mild	Mixed
Easily identifiable	10	10	Mild	Single^1^
Scant	6	0	Moderate	Single
Possible	5	2	Mild	Mixed

^1^Paracetamol intoxication was considered a contributory cause of death

### Other intoxications

The substances involved in the intoxications in control group 1 (other intoxications) were mostly opioids, benzodiazepines, hypnotics, sedating antihistamines, anti-depressants and neuroleptics in various combinations or as single drugs. However, two cases were single intoxications with metformin and one with insulin. In three cases, ethanol was part of a mixed intoxication.

### BHB

Beta-hydroxybutyrate (BHB) had been analysed in femoral blood in 10 cases. Only four cases of these proved positive. One case had a BHB result of 500 µg/g in femoral blood, a case of easily identifiable BFTV with 10 BFTV-positive fields and 10 fields with BFTV-indicative formalin pigment deposition. One case belonged to the group other intoxications with a BHB concentration in femoral blood of 590 µg/g, easily identifiable BFTV and 10 BFTV-positive fields but no BFTV-indicative formalin pigment deposition. The other two cases were hangings, one with BHB concentration in femoral blood of 160 µg/g and the other 56 µg/g. The degrees of BFTV were “possible” and “none”, respectively. Among the cases in which BHB was analysed but found to be below the detection limit, three were cases of paracetamol intoxication with easily identifiable BFTV.

### Vitreous glucose concentrations

Vitreous glucose was analysed in a total of 48 cases and in 14 of the paracetamol intoxication cases. The range for all glucose analyses was 0–42 mmol/L, the mean 1.59 mmol/L, and the median 0.4 mmol/L. The second highest glucose result was 4.5 mmol/L and the highest result among the paracetamol cases was 3.3 mmol/L. In a total of 16 cases, no glucose was detected. 42 mmol/L was the only clearly hyperglycaemic result and it was detected in a case in control group 1 (other intoxications). BHB was not analysed in this case. The degree of vacuolisation in this case was “possible” with 6 areas displaying possible vacuolisation. However, it did not display formalin pigment deposition.

### Liver necrosis

In eight cases, some degree of acute liver necrosis was detected. Seven of these cases were cases of paracetamol intoxication and six of these seven cases had easily identifiable BFTV. Details on these eight cases are presented in supplementary Table 1.

### PAS stain

Seven cases displayed PAS-positivity. Of these, five were equivocal cases with only one PAS-positive field each. One case displayed two PAS-positive fields. Only one case displayed clear findings of PAS-positivity. This was a person with known AUD but no known diabetes, who had been brought to the emergency department after an intentional overdose with paracetamol and who died several days later after treatment in the intensive care unit (ICU) with liver necrosis and liver failure due to paracetamol intoxication. The cause of death was deemed single intoxication with paracetamol based on the background information and hospital records. However, even though the case displayed findings of PAS-positivity, it also displayed easily identifiable BFTV with 10 positive fields and with apparent formalin pigment deposition (see figure S1 in the supplementary material).

### Ethanol

Ethanol in femoral blood was analysed in 67 cases. The mean detected ethanol concentration among these was 0.36‰ and the range was 0.0–2.48‰. Eleven cases had an ethanol concentration ≥ 1.0‰, of which eight belonged to control group 2 (hangings), two were paracetamol intoxications, and one case represented control group 1 (other intoxications).

### Alcohol use disorder and diabetes

Of the paracetamol intoxications, nine had known AUD, one had both known AUD and known diabetes, and two had known diabetes without AUD. In the other intoxication group, seven had known AUD, and three had diabetes. In the hanging group, seven had known AUD, and one had diabetes. In the control groups, none had both AUD and diabetes.

### Easily identifiable BFTV cases

A total of 18 cases displayed “easily identifiable” BFTV. Of these, three belonged to control group 1 (other intoxications), and 15 were cases of paracetamol intoxications. Of the three cases belonging to control group 1, one represented a case of single intoxication with metformin, and had a BHB concentration of 590 µg/g, which could explain the BFTV. The other two cases were a mixed intoxication with pregabalin and zopiclone with three BFTV-positive fields and seven fields with BFTV-indicative formalin pigment deposition, and a single intoxication with alprazolam with four BFTV-positive fields but no fields with BFTV-indicative formalin pigment deposition.

## Discussion

The purpose of this study was to investigate a possible association between BFTV and paracetamol intoxication. We studied kidney samples from 24 cases of paracetamol intoxication and compared the results to 24 cases of other intoxications and 24 cases of hanging. We estimated the occurrence of BFTV in the kidney samples both according to an ordinal scale (degree of BFTV) and a quotient scale (number of BFTV-positive fields). We detected a significant association between paracetamol intoxication and BFTV for both scales. This association has not previously been studied or described.

### Paracetamol intoxication and ketoacidosis

As mentioned above, BFTV has previously been associated mostly with ketoacidotic conditions. Our results indicate that BFTV also occurs in intoxications with paracetamol. Paracetamol intoxication does not cause ketoacidosis, but it may cause lactic acidosis, and a less-well know form of acidosis called 5-oxoproline acidosis or pyroglutamate acidosis [23–25]. BHB was analysed in a very limited number of the cases in our study (only four of the paracetamol intoxications), and in only one of the paracetamol intoxications was BHB pathologically elevated [26–28]. Since September 2017, BHB has been analysed within the Swedish National Board of Forensic Medicine in all cases in which a *post-mortem* sample displays an acetone concentration above the detection level (0.02 mg/ml). This is a reliable method for detecting elevated BHB concentrations [29]. Thus, the cases of paracetamol intoxication in general do not display elevated concentrations of BHB, and the development of BFTV in these cases requires another explanation than ketoacidosis. Neither do our results point to acute alcohol intoxication being a factor in our cases of paracetamol intoxication.

### The post-mortem interpretation of the histological samples

The *post-mortem* interpretation of microscopic kidney samples is challenging, as the tubular cells degrade rapidly after death [12]. Acute tubular necrosis, which may occur in paracetamol intoxication, is difficult to discern from autolysis [30, 31]. Therefore, we did not assess whether signs of acute tubular necrosis were present in our samples. Since this was a retrospective study, we were not able to use fat stain to confirm that the observed vacuoles were fat. Fat stain should be applied before a tissue is paraffin embedded, because during the process of preparing paraffin blocks, the fat is removed and only the vacuolic form of the fat remains, and thus, the fat itself can no longer be stained [32]. However, our results clearly indicate that the vacuoles were not PAS-positive, i.e., they were not glycogen (Armanni-Ebstein-lesions).

Formalin pigment deposition has previously been used as an indicator for fat [12, 13], and we also studied the presence of formalin pigment deposition indicative of BFTV in the kidney samples. We detected a significant correlation between the number of fields with BFTV and the number of fields with BFTV-indicative formalin pigment deposition, and between the degree of BFTV and the number of fields with BFTV-indicative formalin pigment deposition. However, there was no statistically significant correlation between the number of fields with BFTV-indicative fields and the degree of putrefaction, and only a weak correlation between the degree of putrefaction and the general occurrence of formalin pigment deposition. Artefactual formalin pigment depositions in histological preparations result from an interaction between formalin and haem. The depositions occur primarily in acidic environments [33–35], and they are especially common in decomposed bodies [33, 36], although research on the subject has been limited [33]. However, formalin pigment appears to have a particular affinity for fat [12, 37]. It has been suggested that this could be due to a breakdown of triglycerides into fatty acids through hydrolysis that occurs in the decomposition process [12, 38]. Fatty acids, unlike triglycerides, would provide an acidic environment suitable for formalin pigment deposition. Thus, a certain degree of putrefaction is required for the development of formalin pigment deposition, but unless there is fat in the samples to begin with, the formalin will have no fatty acids to attach to, and putrefaction alone does not cause BFTV-indicative formalin pigment deposition. Since our results showed that there was no statistically significant correlation between BFTV-indicative formalin pigment deposition and the degree of putrefaction, this serves as an indication that our BFTV-indicative formalin pigment depositions were not merely a result of putrefaction, and that the observed vacuoles indeed constitute fat.

For the evaluation of BFTV, we employed a double approach. We made an overall evaluation according to the scale “negative-possible-scant-easily identifiable”, a scale which has been used previously [7]. In addition, we adopted a semi-quantitative estimation by marking ten fields in each slide and counting the number of fields with tubules that displayed vacuoles consistent with BFTV. No minimum number of vacuoles in the tubular cells was required in a field for it to be judged positive. As the fields were marked randomly, it was possible that few or no fields displayed vacuoles, but based on an overall estimation of the sample, it was still considered “scant” or “easily identifiable” concerning BFTV. This was due to the somewhat random pattern of BFTV. In a few samples, no or very few marked fields were positive for vacuoles, yet because of the existence of obviously positive areas elsewhere in the sample, the overall assessment was still “scant” or “easily identifiable”. However, the number of positive fields usually correlated with the degree of BFTV.

### Kidney physiology and the toxicity of paracetamol

The kidneys are one of the organs in the body with the highest demand for energy, and of the kidney cells, the proximal tubular cells (PTCs) are the ones with the highest level of energy consumption. Because of this high energy demand, the PTCs gain their energy (adenosine triphosphate, ATP) from beta-oxidation of FFA, i.e., fatty acid oxidation (FAO). The FAO mainly occurs in the mitochondria and it is a process with a high demand for oxygen, which renders the PTCs vulnerable to ischaemia. The PTCs have a limited capacity for glycolysis but can switch to glycolysis when FAO is inhibited, for example when the oxygen supply is insufficient [39–41]. The FFA are transported into the PTCs primarily on albumin and to a lesser extent as unbound FFA, both from the basolateral side and from the tubular lumen [40]. If a surplus of FFA is transported to the kidney in proportion to its energy needs, the excess FFA can be esterified with glycerol to form intracellular lipid droplets in the form of triglycerides. These droplets can vary in size from < 1 micrometre in range to droplets that can be detected by microscopy with special fat stain [40]. A decrease in the FAO causes an accumulation of lipids in the cytoplasm [39–41], as does an increased concentration of FFA in serum [42]. Such lipid accumulation presumably is important both in acute kidney injury and in chronic kidney diseases, and has been referred to as lipotoxicity [39, 40, 43]. It has been suggested that the PTCs’s ability of simultaneous apical and basolateral uptake of FFA, in combination with an increased FFA synthesis, and/or diminished FAO, could lead to intracellular fat accumulation in the PTCs [44, 45]. The triglycerides appear to act as a fat storage, whereas the FFA and their products exert a toxic effect [46–48]. The triglyceride storage of excess FFA may even serve as protective measure against lipotoxicity and subsequent cell death, at least initially [47, 49]. Furthermore, FFA accumulation causes mitochondrial dysfunction, which in turn leads to further FFA accumulation [43]. Of the kidney cells, the tubular cells, and the PTCs especially, are the cells that are primarily affected by various stressors [41]. A number of physiological stressors, including ischaemia, toxins, and obstructive and immunological insults have been described to cause fatty accumulation in the tubules [45]. It has been suggested that the triglyceride accumulation in the PTCs in fact represents a general “stress response” of the kidneys, involving many different, complex cellular pathways [45, 50].

The toxic effects of paracetamol include ones that are relevant in the development of BFTV. Paracetamol intoxication may cause both hypoglycaemia and hyperglycaemia [23, 24, 51–56]. Establishing hypoglycaemia *post mortem* is not possible without corroborating findings [57, 58]. It is therefore not possible to judge whether any of the subjects in our study suffered from hypoglycaemia at the time of death. However, our findings do not point to hyperglycaemia in any of the paracetamol intoxications. In any case, hypoglycaemia increases the amount of FFA in the circulation through the release of catecholamines [59]. This could be part of the explanation for BFTV in paracetamol intoxication.

Another important aspect of paracetamol toxicity from the point of view of BFTV is the renal toxicity. Even though the exact mechanism of the toxic effect of paracetamol on the kidney is less well known than on the liver, it seems that paracetamol in overdose exerts a toxic effect on the kidney mitochondria as well [60, 61]. Since the FAO occurs in the mitochondria, impaired mitochondrial function will decrease the use of FFA and increase the availability of FFA for triglyceride accumulation [50].

Paracetamol intoxication may also cause the ischaemia that is part of the development of BFTV. Studies on mice have implicated that in paracetamol toxicity, a hepatic congestion develops. This congestion is enough to cause severe entrapment of red blood cells in the liver. This removal of red blood cells from the general circulation may lead to hypovolaemia, hypotension, and shock [62]. Thus, the hepatic congestion may cause hypovolaemia in paracetamol intoxication, and thus, may cause ischaemia, even though the liver volume in mice is larger in proportion to body size than in humans, and the results therefore cannot be directly transferred to humans.

In conclusion, BFTV develops due to an accumulation of triglycerides in the PTCs in the form of fat droplets (vacuoles) in conditions with elevated levels of blood FFA in combination with a reduced oxygen supply to the kidneys or an inability of the PTC mitochondria to exert FAO. Paracetamol intoxication may impair the mitochondrial function in the PTCs, which reduces the capacity to oxidise FFA and causes an increased availability of FFA due to hypoglycaemia, and which may cause hypovolaemia and hypotension, resulting in renal ischemia and a further decrease in the capacity to oxidise FFA.

### Limitations

The fact that this study was retrospective is in itself a limitation. The retrospective nature also hindered the use of fat stain, which would have made our observations more reliable. The inter-rater reliability was moderate. Based on the results, it appeared that one of the evaluators was more hesitant overall to deem a finding as BFTV. However, both evaluators were able to differentiate the groups, to which they were blinded, indicating that the evaluation methods that were used in the study were useful. The microscopic evaluation of *post-mortem* kidney samples is challenging due to the degradation of the tissues. The evaluation of BFTV and of the pattern of formalin pigment deposition was obviously subjective, something that is true for many histopathological studies. However, we have evaluated the kidney samples for BFTV in our routine work at our department for years and stained samples with osmium when necessary. It is our experience that with time, it is possible to develop a good sensitivity for BFTV even in HE-stain and even with moderate putrefaction present. We consider that the measures taken in our study, i.e., blinding the samples, considering the degree of putrefaction, and the statistical analyses, compensate for the subjective nature of the evaluation of the samples.

## Conclusions

Our study shows that paracetamol intoxication is associated with the development of BFTV, a phenomenon that in existing literature is almost exclusively associated with ketoacidotic conditions. A pathophysiological explanation of the development of BFTV in paracetamol intoxication is proposed and the discovery offers a perspective that has not previously been described. BFTV is a consequence of metabolic imbalances and ischaemia, and probably exists in a variety of conditions besides paracetamol intoxication and ketoacidosis. BFTV is thus not specific for ketoacidosis, as has previously sometimes been suggested.

## Supplementary Information

Below is the link to the electronic supplementary material.

Supplementary Material 1

## Data Availability

The assessment data that support the findings of this study are available upon reasonable request from the corresponding author.
