# Supplementary material for: Paracetamol (acetaminophen) intoxication and basal fatty tubular vacuolisation: a case-control study and review of the literature
Source: Int J Legal Med. 2026 Aug 1;140(6):4277–87. doi: 10.1007/s00414-026-03942-7 (PMC13658482; doi:10.1007/s00414-026-03942-7)
Supplement: Supplementary file 1 [file 414_2026_3942_MOESM1_ESM.docx]

Supplementary table 1. Cases with liver necrosis

| Category | Degree of BFTV | Number of BFTV- positive fields | General presence of formalin pigment deposition | Number of fields with formalin pigment deposition | Degree of putrefaction |
| --- | --- | --- | --- | --- | --- |
| Paracetamol intoxication | Easily identifiable | 10 | No | 2 | Moderate |
| Other intoxication | Possible | 4 | No | 0 | Minimal |
| Paracetamol intoxication | Scant | 6 | No | 6 | Slight |
| Paracetamol intoxication | Easily identifiable | 10 | No | 10 | Minimal |
| Paracetamol intoxication | Easily identifiable | 5 | No | 4 | Moderate |
| Paracetamol intoxication | Easily identifiable | 10 | No | 10 | Slight |
| Paracetamol intoxication | Easily identifiable | 6 | Yes | 8 | Moderate |
| Paracetamol intoxication | Easily identifiable | 10 | No | 0 | Moderate |

Notes: BFTV = basal fatty tubular vacuolisation.

**Supplementary fig. 1** The light pink arrow points to PAS-positive material in the tubuli, possibly glycogen. The purple arrow points to BFTV, which is notable also in this PAS-stain. BFTV = Basal fatty tubular vacuolisation. PAS = Periodic acid-Schiff stain.
